# Treatment of Candida nivariensis Blood Stream Infection With Oral Isavuconazole

**DOI:** 10.7759/cureus.32137

**Published:** 2022-12-02

**Authors:** Olivia Randazza, Kallie Erickson, Travis Denmeade, Vera Luther, Elizabeth Palavecino, James Beardsley

**Affiliations:** 1 Department of Pharmacy, Atrium Health Wake Forest Baptist, Winston-Salem, USA; 2 Department of Internal Medicine, Wake Forest University School of Medicine, Winston-Salem, USA; 3 Department of Pathology, Wake Forest University School of Medicine, Winston-Salem, USA

**Keywords:** fungemia, candidemia, isavuconazonium, isavuconazole, candida nivariensis

## Abstract

*Candida nivariensis *is a rarely isolated yeast that is now considered a species within the *Candida glabrata* complex. Anti-fungal susceptibilities and treatments of *Candida nivariensis *are often assessed on a case-by-case basis. In this case, a 70-year-old male with a complex medical history presented to a large academic medical center in the United States for vascular surgery. After surgery, the patient’s white blood cell count increased prompting an infectious workup. The patient was found to have a *Candida nivariensis* bloodstream infection of unknown origin. Given the patient’s clinical stability and QTc prolongation, he was treated with a 14-day course of oral isavuconazole. The patient experienced resolution of symptoms and clearance of subsequent blood cultures. At the time of writing this case report (11 months later), he has had no relapse of his fungal infection. Based on a search of the medical literature, this appears to be the first published case of *Candida nivariensis* fungemia successfully treated with oral isavuconazole.

## Introduction

The yeast, *Candida nivariensis*, is phenotypically similar to *Candida glabrata* [1-3]. *C. nivariensis* is identified through the use of genotypic measures such as multiplex polymerase chain reaction (PCR) or matrix-assisted laser desorption/ionization-time of flight (MALDI-TOF) mass spectrometry which most accurately distinguishes it from other members of the *C. glabrata* complex (e.g., *C. glabrata* and *Candida bracarensis*) [1-3]. In a review of previous reports containing 130 isolates of *C. nivariensis*, the fluconazole minimum inhibitory concentration (MIC) varied widely from 0.06 µg/mL to >256 µg/mL [1]. Similar to *C. glabrata*, *C. nivariensis* is often resistant or susceptible-dose dependent to fluconazole [1-3]. Sources of isolated *C. nivariensis* include blood, vaginal secretions, urine, oropharyngeal secretions, cerebrospinal fluid, ascitic fluid, nails, and bronchial-alveolar lavage (BAL) fluid [1-3].

Isavuconazole is a second-generation triazole shown to be effective in vitro against *Candida* species, including *C. glabrata* [4,5]. The 2019 ACTIVE trial for isavuconazole failed to demonstrate non-inferiority for the primary endpoint of overall response, defined as mycological eradication and clinical cure or improvement, when compared to caspofungin followed by oral voriconazole for candidemia or invasive candidiasis [6]. Notably, 11% (43/400) of cultures in this trial isolated *C. glabrata*. While isavuconazole was inferior for the primary outcome, the overall success rate for oral step-down therapy was higher in the isavuconazole arm compared to the caspofungin/voriconazole arm (82.6% vs. 77.5%) which may lend toward using isavuconazole as an oral step-down agent in candidemia [4,6]. Isavuconazole has a mild side effect profile including nausea, vomiting, and diarrhea. A key adverse effect is hepatotoxicity, although less than other triazoles. However, a benefit of isavuconazole is that it is associated with QTc shortening, rather than QTc prolongation, as is seen with most other triazoles [4].

There are no published reports on the treatment of *C. nivariensis* with isavuconazole in the English medical literature. Only two studies were found that reported isavuconazole susceptibility testing for *C. nivariensis* [7,8]. Sharma et al. performed in vitro testing of five isolates (one BAL and four vaginal samples), using the Clinical and Laboratory Standards Institute (CLSI) broth microdilution method per the M27 guidelines, and found isavuconazole exhibited the most potent activity of all antifungals tested (MIC range: 0.015-0.25 µg/mL, geometric mean: 0.03 µg/mL) [7]. In another study of *C. nivariensis* oropharyngeal candidiasis in an Indonesian patient with human immunodeficiency virus infection, the MIC for isavuconazole was 0.008 µg/mL [8]. We describe a case of *C. nivariensis* bloodstream infection successfully treated with oral isavuconazole at a large, academic medical center in the United States.

## Case presentation

A 70-year-old male was admitted to the hospital’s vascular surgery service with a three-month history of non-healing right foot wounds. The patient’s past medical history was significant for insulin-dependent diabetes mellitus type 2, previous tobacco use, atrial fibrillation, coronary artery disease with prior coronary artery bypass graft, heart failure with preserved ejection fraction, chronic obstructive pulmonary disease, and end-stage renal disease on peritoneal dialysis (PD). Additionally, he had a deceased donor renal transplantation, which failed after 13 years due to the development of skin cancers requiring a reduction of immunosuppression. In the setting of chronic headaches and temporal pain, he was also started on prednisone 60 mg daily by his primary care provider for possible temporal arteritis approximately one week prior to admission and a left-sided temporal artery biopsy was subsequently performed.

On the day of admission, he underwent revascularization of the right lower extremity including a femoral endarterectomy and femoral artery to posterior tibial artery bypass followed by bedside debridement of a necrotic right plantar wound down to a healthy, bleeding base by podiatry. Five days post-revascularization, the patient was noted to have an increase in white blood cell count from 19.6 to 24.1 x 10^3^/µL with symptoms of dysuria and increased urinary frequency. Urinalysis revealed positive leukocyte esterase, and intravenous (IV) ceftriaxone 1 g daily was empirically initiated for a complicated urinary tract infection (UTI). The patient’s urine culture grew > 100,000 CFU/mL of pan-susceptible *Pseudomonas aeruginosa,* and ceftriaxone was changed to piperacillin-tazobactam 3.375 mg IV every 12 hours. The patient remained on piperacillin-tazobactam for two days and was then transitioned to oral ciprofloxacin 500 mg daily.

Despite antibiotic treatment for a UTI, his leukocytosis worsened and peaked at 40.8 x 10^3^/µL nine days after surgery. A peritoneal fluid analysis and culture was obtained to assess for peritonitis; however, it was negative for leukocytes and there was no growth on culture. Of note, the patient had serous drainage from his surgical incision site, but there was no evidence of acute infection. The temporal artery biopsy results were negative for temporal arteritis, and a steroid taper was initiated.

Concurrently, the infectious diseases (ID) service was consulted nine days after admission, in the setting of leukocytosis of unclear etiology. ID recommended obtaining blood cultures and computerized tomography (CT) imaging of the chest, abdomen, pelvis, and thigh. This imaging revealed a moderate volume of abdominopelvic ascites; stable, nonspecific stranding about the transplanted kidney; and postsurgical changes in the thigh. No abscesses or clear signs of infection were identified. 

Two sets of blood cultures taken two days apart returned positive for yeast that was originally concerning for possible dimorphic fungi. As the patient was hemodynamically stable, the decision was made to use a triazole antifungal rather than amphotericin B. Voriconazole 450 mg (6 mg/kg of ideal body weight) for two doses was initiated empirically. After one dose of voriconazole, the patient was found to have a prolonged QTc interval of 555 ms on an electrocardiogram (EKG). The patient was then switched to isavuconazole 372 mg by mouth every eight hours for six doses followed by 200 mg by mouth daily. Ciprofloxacin was also changed to cefepime in light of QTc prolongation, and his complicated UTI was treated for a total of eight days. The patient’s hospital course was further complicated by non-ST-elevation myocardial infarction (NSTEMI) which was medically managed.

A thorough workup was pursued for the source of the patient’s fungemia. Ophthalmology identified no evidence of fungal eye disease. Peritoneal fluid cell count was again unremarkable, and a fungal smear and culture were negative. A chest x-ray showed no acute pulmonary processes, and a magnetic resonance imaging (MRI) of the right foot was negative for abscess or osteomyelitis. The patient had no indwelling central lines, and blood cultures were drawn peripherally. Additionally, dermatology was consulted to evaluate a lesion on the patient’s left index finger. They had low concern for cutaneous infection, and a skin biopsy was not obtained. A transthoracic echocardiogram (TTE) showed no signs of infectious vegetation on the heart. The renal transplant team was consulted to further investigate the stranding around the transplanted kidney identified by CT imaging, and given its stable appearance compared to prior imaging, they recommended continued observation over renal biopsy. While the patient had several risk factors for candidemia, a discrete source remained unclear.

The yeast organism recovered from the blood cultures was identified as *C. nivariensis* by MALDI-TOF mass spectrometry (MS) (Bruker Biotyper). The clinical microbiology laboratory identified MIC values for 5-fluorocytosine, amphotericin B, fluconazole, and itraconazole through the use of the Thermo Fisher Yeast-One™ (Waltham, Massachusetts, USA) broth microdilution panel as seen in Table 1. A special MIC for isavuconazole was requested for the *C. nivariensis* isolate from the University of Texas (UT) Health San Antonio Fungus Testing Laboratory, which resulted after the patient’s discharge. UT Health San Antonio used the CLSI broth microdilution method per the M27 guidelines to determine the isavuconazole MIC [9].

**Table 1:** Drug susceptibility testing against isolated C. nivariensis The isavuconazole minimum inhibitory concentration (MIC) was obtained from the University of Texas (UT) Health San Antonio Fungus Testing Laboratory using broth microdilution. The MICs for all other antifungals were obtained from the case institution using broth microdilution.

Drug	MIC (µg/mL)
5-Fluorocytosine	0.25
Amphotericin B	1
Fluconazole	4
Itraconazole	0.5
Isavuconazole	0.125

The patient’s hospital course was complicated by a period of hypotension, requiring a brief stay in the surgical intensive care unit. The patient was stable for the rest of his hospital stay. After 21 days, he was discharged home with isavuconazole 372 mg by mouth daily for an additional seven days to finish out a total of 14 days of therapy from the first negative blood culture. There has been no evidence of a relapse of his fungal infection in the 11 months following hospital discharge.

## Discussion

We report a case of *C. nivariensis* fungemia successfully treated with isavuconazole. To our knowledge, this is the first reported case. The special isavuconazole MIC obtained in this case from UT Health San Antonio of 0.125 µg/mL is similar to and falls within the MIC range of 0.015-0.25 µg/mL for the *C. nivariensis* isolates from vaginal and bronchial specimens reported in Sharma et al. [7]. An elevated fluconazole MIC of 4 µg/mL with lower MICs to other triazoles, like itraconazole and isavuconazole, is similar to susceptibility patterns seen in other case reports [1-3]. MIC interpretations are not available as no established breakpoints for *C. nivariensis* have yet been published from the CLSI in the M60 standards [10]. The European Committee on Antimicrobial Susceptibility Testing (EUCAST) reports anti-fungal non-species-related breakpoints for *Candida* which lists an MIC of 2 mg/L as susceptible and an MIC of 4 mg/L as resistant in regards to fluconazole [11]. Given similarities in phenotype, MICs reported for *C. glabrata* could be considered for comparison. EUCAST reports an MIC of ≤ 0.001 mg/L as fluconazole susceptible and an MIC of > 16 mg/L as fluconazole resistant for *C. glabrata* [11]. CLSI reports an MIC ≤ 32 µg/mL to be fluconazole susceptible-dose dependent and an MIC of ≥ 64 µg/mL to be resistant to fluconazole for *C. glabrata* [10].

## Conclusions

Given the successful outcome described in this case report, isavuconazole may be considered as a future oral option for the treatment of *C. nivariensis* infections in similar patients. Given the paucity of literature on *C. nivariensis*, additional studies should describe the identification methodologies, MIC breakpoints, and treatment outcomes of *C. nivariensis* infections.
